# Comparison of [^18^F]FIMP, [^11^C]MET, and [^18^F]FDG PET for early-phase assessment of radiotherapy response

**DOI:** 10.1038/s41598-023-29166-y

**Published:** 2023-02-03

**Authors:** Satoshi Nozaki, Yuka Nakatani, Aya Mawatari, Nina Shibata, William E. Hume, Emi Hayashinaka, Yasuhiro Wada, Hisashi Doi, Yasuyoshi Watanabe

**Affiliations:** 1 Laboratory for Pathophysiological and Health Science, RIKEN Center for Biosystems Dynamics Research, 6-7-3 Minatojima Minamimachi, Chuo-Ku, Kobe, Hyogo 650-0047 Japan; 2 Novel PET Diagnostics Laboratory, RIKEN Innovation Center, Kobe, Hyogo Japan; 3 Laboratory for Labeling Chemistry, RIKEN Center for Biosystems Dynamics Research, Kobe, Hyogo Japan

**Keywords:** Cancer imaging, Radiotherapy

## Abstract

Several limitations of [^18^F]FDG have been reported, such as nonspecific uptake of inflammation foci. Moreover, [^11^C]MET has been found to accumulate in normal and inflammatory tissues as well as tumors. To increase specificity to tumor tissues, PET probes with tumor-specific molecular targets have been actively developed. [^18^F]FIMP was found to be highly accumulated in LAT1-positive tumors but not in inflamed tissue. The aim of this study was to explore whether [^18^F]FIMP can be used for the early-phase evaluation of radiotherapy accompanied by inflammation, and compare its effectiveness with those of [^11^C]MET and [^18^F]FDG. Tumor uptake of [^18^F]FIMP decreased at day 1 after irradiation, and remained low until day 14. Comparatively, that of [^18^F]FDG initially decreased at day 3 but was transiently elevated at day 7 and then decreased again at day 10. Decreased tumor uptake of [^11^C]MET was observed at day 10. In line with the uptake of [^18^F]FIMP, the ratio of Ki-67 immuno-positive cells in tumor tissues significantly decreased at day 1, 7, and 10 as compared with that in the control. These findings suggest that [^18^F]FIMP may be a PET probe involved in the early detection and prediction of radiotherapy efficacy, although further clarification is needed.

## Introduction

Radiotherapy is used to treat a wide variety of cancers with more than 50% of all cancer patients^1^. Detection of anatomical changes using computed tomography (CT) and magnetic resonance imaging (MRI) after radiotherapy has commonly been used for evaluation of the therapeutic effect. The current clinical standard for assessing responses to treatment is to determine the reduction in tumor size by CT and/or MRI using the response evaluation criteria in solid tumors (RECIST) clinical guideline^2^. However, changes in tumor size have been found to be very slow requiring several weeks or months after radiotherapy^3^. Moreover, RECIST criteria focus only on reducing tumor size^4^ and not necessarily correlating the result with patient survival^5–7^.

Chemo- and radio-therapy bring about biochemical changes in tumor tissues which occur before the size reduction of tumor. It can be determined by highly sensitive PET, not CT or MRI. Recently, novel clinical criteria have been proposed for assessing tumor volume change including biological information obtained from PET imaging of glucose metabolism using 2-deoxy-2-[^18^F]fluoro-D-glucose (FDG), called PET response criteria in solid tumors (PERCIST)^2^. However, [^18^F]FDG is actively transported into cells via glucose transporters, which are expressed not only in tumor tissue but also in inflammatory foci, where they regulate glucose metabolism to promote inflammatory responses^8,9^. Glucose transporters are also expressed in cells of the central nervous system, which limits imaging of brain tumors^10^. Hence, [^18^F]FDG PET cannot distinguish between tumor tissue and inflamed lesions^11^, and therefore results in false-positives for tumor diagnosis^12^.

To overcome the disadvantages of [^18^F]FDG, radiolabeled amino acid PET probes such as L-[methyl-^11^C]methionine (MET) were developed^13^. MET is a natural analogue of methionine that is incorporated into proteins, and its incorporation probably suggests enhanced protein synthesis and is useful in predicting the histologic grade of astrocytoma^14^. However, [^11^C]MET has been found to accumulate in normal and inflammatory tissues as well as tumors^15–17^. To increase specificity to tumor tissues, PET probes with tumor-specific molecular targets have been actively developed.

L-type amino acid transporter 1 (LAT1) is one of the sodium-independent L-type amino acid transporters^18^. LAT1 is highly expressed in a variety of human tumors and is a promising target for both imaging and therapy^19^. Several PET probes targeting LAT1 have been reported, including [^18^F]fluoro-ethyl-tyrosine (FET)^20^, and 3-[^18^F]fluoro-α-methyl-L-tyrosine (FAMT)^21^. However, none of them are widely used, probably because the signal/noise ratio is not sufficient.

Recently, we also designed and synthesized a novel α-methyl-L-phenylalanine derivative to specifically target LAT1, (*S*)-2-amino-3-[3-(2-[^18^F]fluoroethoxy)-4-iodophenyl]-2-methylpropanoic acid ([^18^F]FIMP). [^18^F]FIMP showed high accumulation in the tumor tissues and low accumulation in inflamed tissues in tumor-bearing mice^22^ and the patient with glioblastoma^23^.

Radiotherapy mainly elicited strong inflammation, which sometimes gave false positive results with [^18^F]FDG. Since LAT1 is a tumor-specific transporter, our LAT1-targeted [^18^F]FIMP may be able to correctly assess the effects of radiotherapy.

Here, we aimed to determine whether [^18^F]FIMP, after reduced accumulation in inflamed tissues, is useful for evaluating the effect of radiation treatment compared to the conventional PET probes [^11^C]MET and [^18^F]FDG.

## Results

### Time-dependent change of tumor volume after irradiation

**Figure 1:**
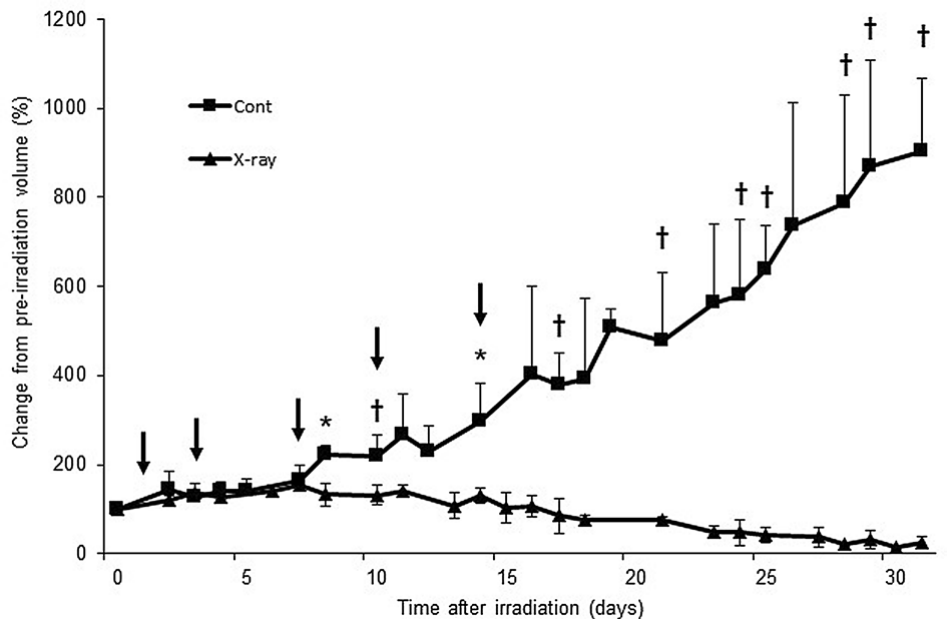
Time-dependent change in tumor volume of LNZ308 tumors after irradiation. Tumors on right paws were irradiated on day 0 at a dose of 60 Gy. Each point represents mean ± SD of the change from pre-irradiation tumor volume (n = 4–6). Probe uptake studies were performed on days 1, 3, 7, 10, and 14 after irradiation, as indicated by arrows. *p < 0.05 and †*p* < 0.01, as compared with control.

The tumor volume in the control group was found to progressively increase until 31 days as compared with the irradiated group [control group, 903.9 ± 163.6; irradiated group, 24.6 ± 15.0 percent changes from pre-irradiation volume (%dVol.)]. However, the tumor volume in the irradiated group did not show a significant decrease until the 7th day after irradiation (control group, 155.1 ± 11.4; irradiated group, 164.1 ± 35.1%dVol.) and significantly decreased from the 8th day after irradiation (control group, 223.6 ± 17.4; irradiated group, 132.2 ± 25.8%dVol.) compared to controls (Fig. 1).

In a comparison of before and after irradiation, the tumor volume in the control group significantly increased from day 7 (164.1 ± 35.1%dVol.) as compared with pre-irradiation (day 0). However, the tumor volume in the irradiated group significantly increased initially until day 11 (139.3 ± 13.4%dVol.), and thereafter it significantly decreased from day 18 (77.1 ± 8.8%dVol.) as compared with pre-irradiation (day 0) (Fig. 1).

### Histological changes in the tumor tissue after irradiation

**Figure 2:**
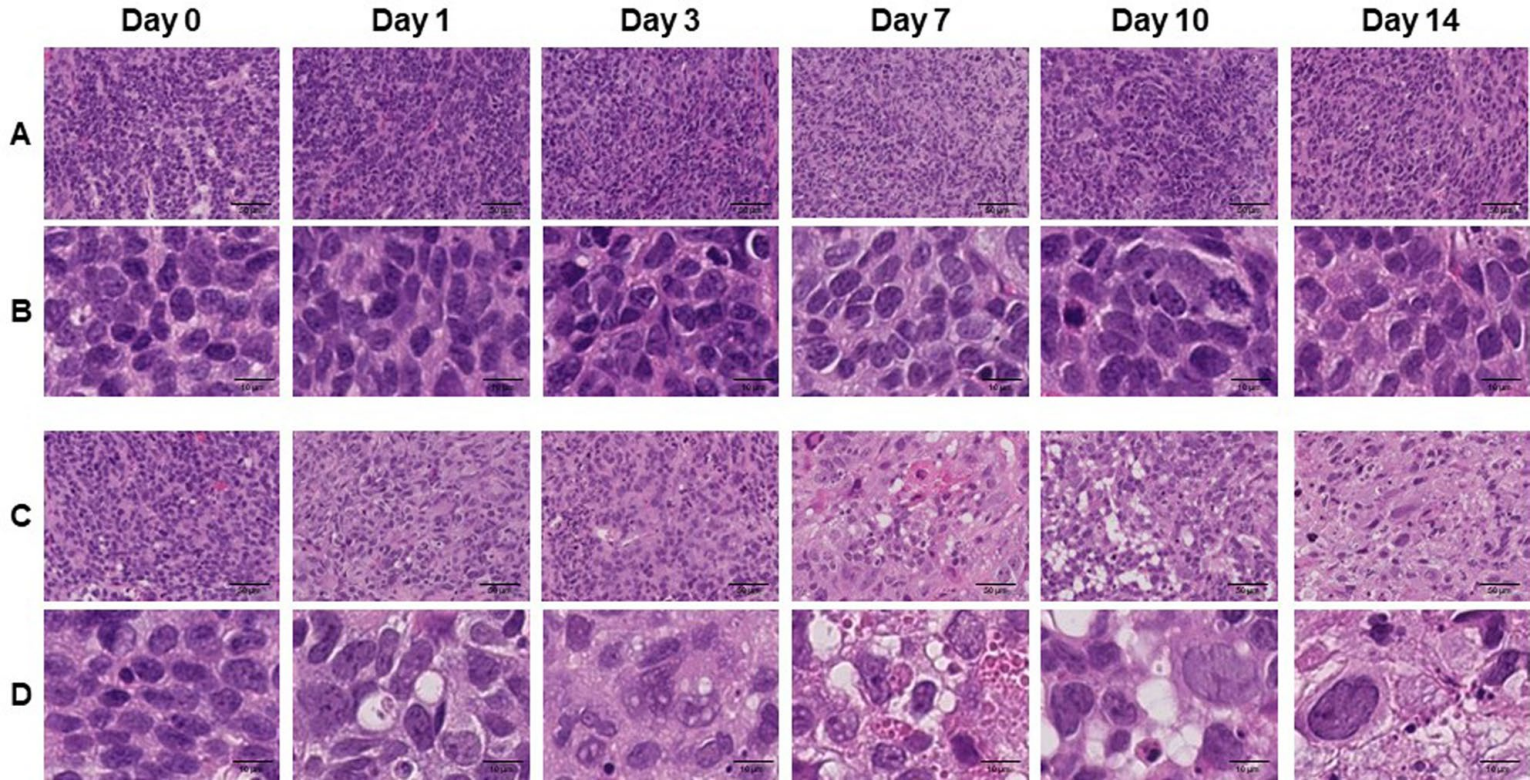
Hematoxylin and Eosin (HE) stained sections of LNZ308 tumors after irradiation. Tumors on right paws were irradiated on day 0 at the dose of 0 Gy (**A** and **B**) or 60 Gy (**C** and **D**). Photomicrographs of low (**A** and **C**) and high (**B** and **D**) magnification show HE stained tumor sections at days 0, 1, 3, 7, 10, and 14 after irradiation (N = 1 each treatment group).

To confirm irradiation-associated changes in tumor tissue, a section of the tumor tissue was stained by H.E. (Fig. 2). Although, time-dependent change was not observed in control tissue, several characteristic changes related to cell death were observed in the irradiated tissue such as nuclear and cytoplasmic vacuolization (day 1 and 3), pyknosis and infiltration of inflammatory cells (day 7), foamy cytoplasm and swelling of nuclei (day 10), and infiltration of fibroblast (day 14) (Fig. 2).

**Figure 3:**
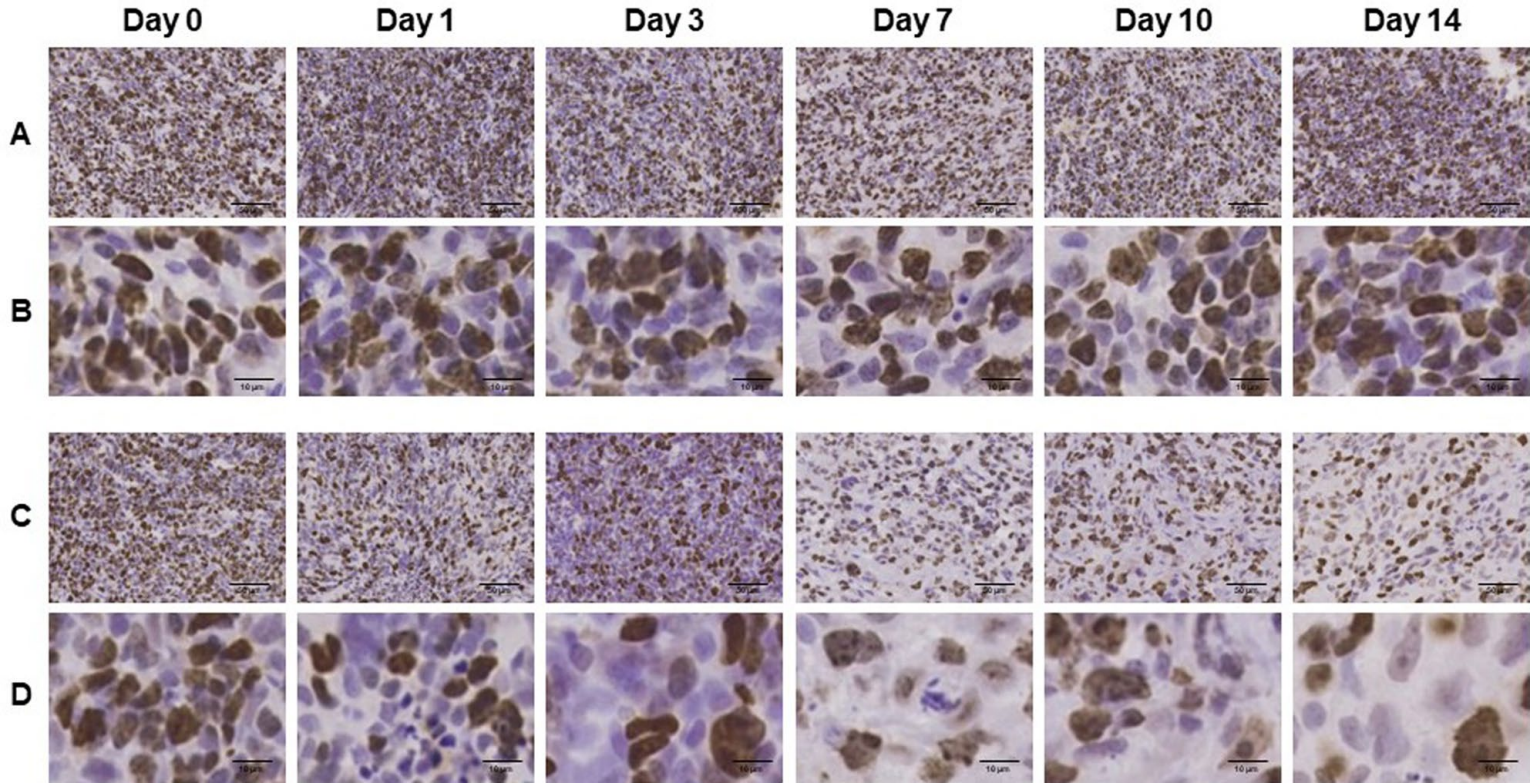
Ki-67 immunohistochemically stained sections of LNZ308 tumors after irradiation. The cell proliferation antigen Ki-67 was used as a marker of cell proliferation. Tumors on right paws were irradiated on day 0 at the dose of 0 Gy (**A** and **B**) or 60 Gy (**C** and **D**). Photomicrographs of low (**A** and **C**) and high (**B** and **D**) magnification. Ki-67 stained tumor sections are shown at days 0, 1, 3, 7, 10, and 14 after irradiation (N = 1 each treatment group).

**Figure 4:**
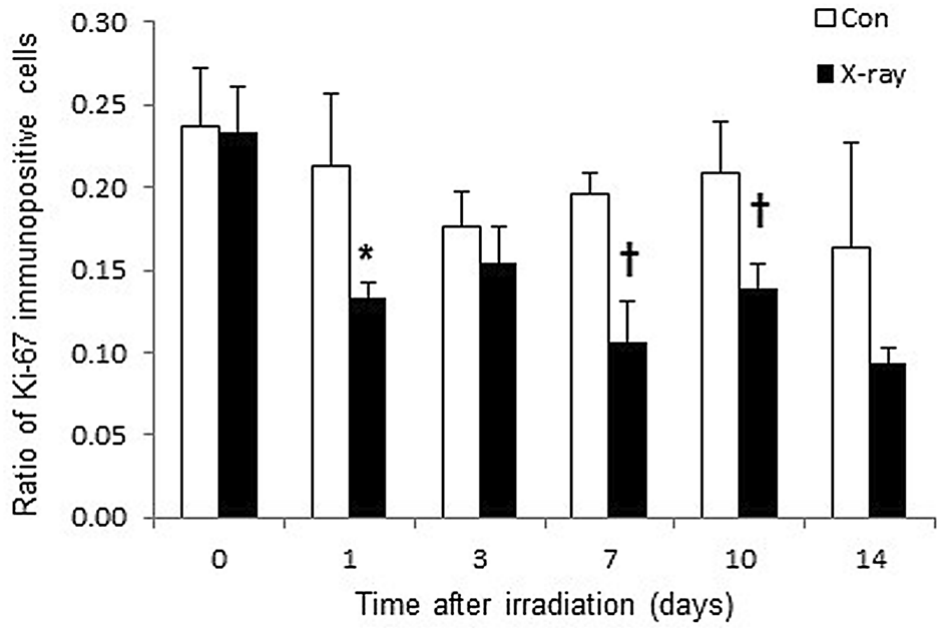
Semi-quantitative analysis of immunohistochemical expression of Ki-67 in LNZ308 tumors after irradiation. Tumors on right paws were irradiated on day 0 at the dose of 60 Gy. Each point represents mean ± SD of the ratio of Ki-67 immunopositive cells (n = 4). *p < 0.05 and †*p* < 0.01, as compared with control.

To confirm irradiation-associated changes in cell proliferation, sections of tumor tissues were stained with anti-Ki-67 antibody (Fig. 3). Ki-67 immunoreactivities decreased in a time-dependent manner after irradiation. Semi-quantitative analysis of immunohistochemical expression of Ki-67 in LNZ308 tumors after irradiation was performed (Fig. 4). The ratio of Ki-67 immuno-positive cells was significantly decreased at days 1, 7, and 10 after irradiation compared to that in the control.

**Figure 5:**
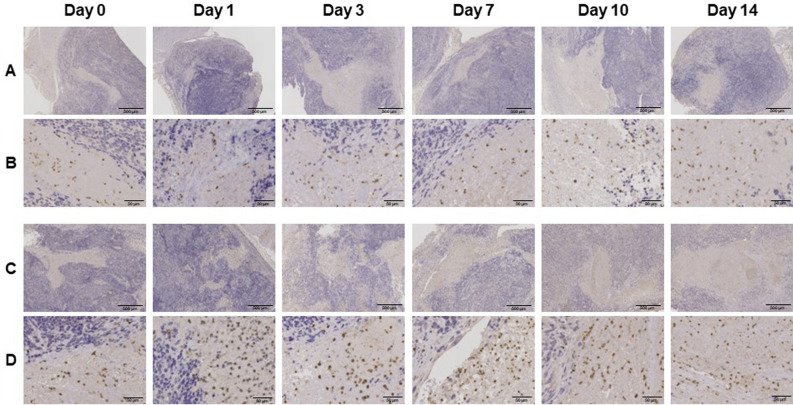
CD11b immunohistochemically stained sections of LNZ308 tumors after irradiation. The integrin alpha chain family member protein CD11b was used as a marker of inflammation. Tumors on right paws were irradiated on day 0 at the dose of 0 Gy (**A** and **B**) or 60 Gy (**C** and **D**). Photomicrographs of low (**A** and **C**) and high (**B** and **D**) magnification reveal CD11b stained tumor sections at days 0, 1, 3, 7, 10, and 14 after irradiation (N = 1 each treatment group).

To confirm the induction of irradiation-associated inflammation, sections of tumor tissues were stained with anti-CD11b antibody (Fig. 5). CD11b immunoreactivities tended to increase 1 day after irradiation compared to those in the control.

### Time-dependent change in PET probe uptake after irradiation

**Figure 6:**
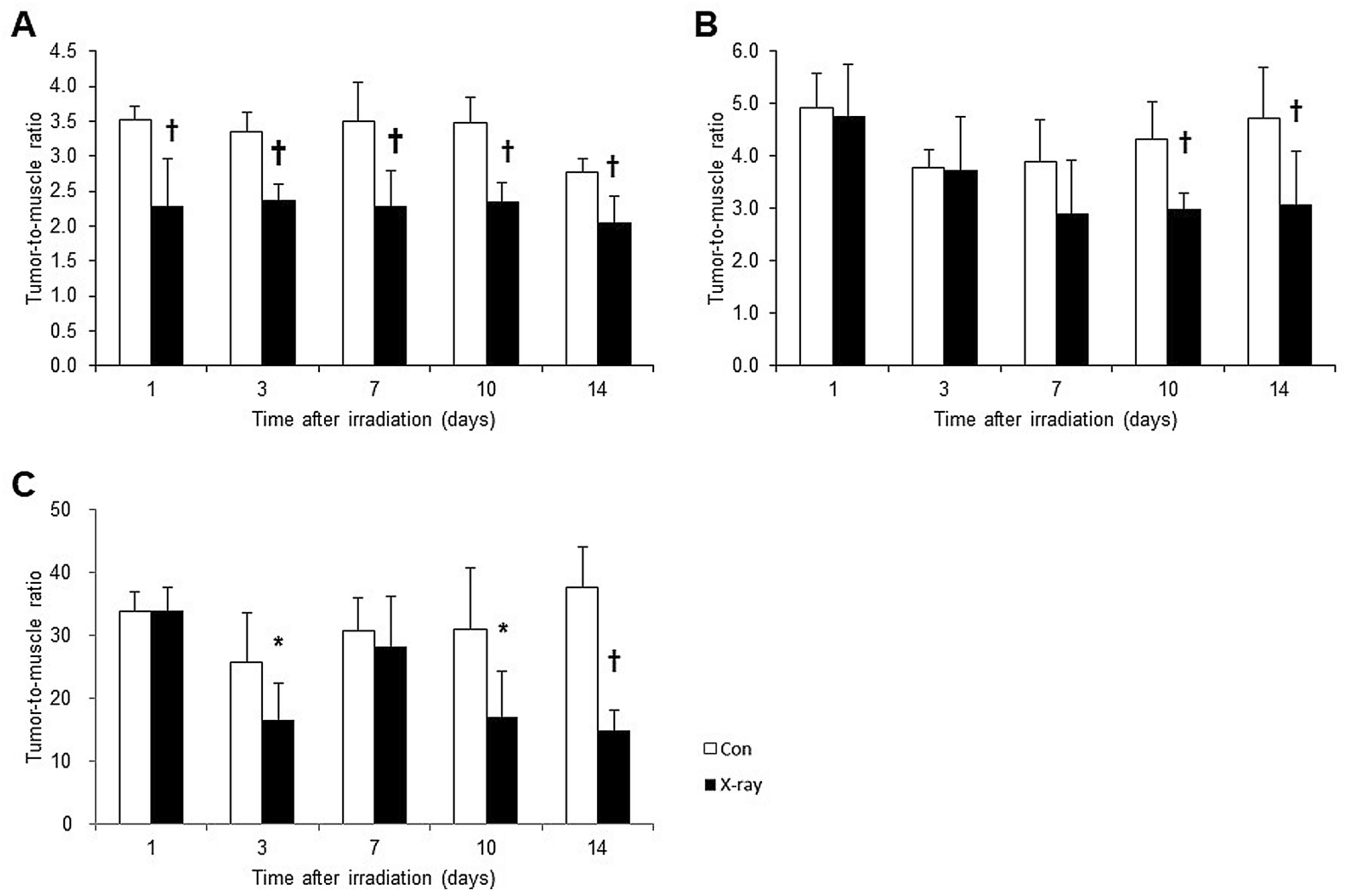
Change in probe uptake of LNZ308 tumors after irradiation. Tumors on right paws were irradiated on day 0 at the dose of 60 Gy. Probe uptake studies were performed at days 1, 3, 7, 10, and 14 after irradiation. Uptake of [^18^F]FIMP (**A**), [^11^C]MET (**B**), and [^18^F]FDG (**C**) were denoted by the tumor-to-muscle ratio (TMR). Each point represents mean ± SD (n = 4–8). *p < 0.05 and †*p* < 0.01, as compared with control.

PET probe accumulations were evaluated in the dissected tissues using the mice model with LNZ308 tumor. [^18^F]FIMP accumulations were significantly decreased from day 1 compared to those in the control (Fig. 6A). However, [^11^C]MET accumulations significantly decreased from day 10 compared to those in the control (Fig. 6B). [^18^F]FDG accumulations were significantly decreased at day 3. However, this accumulation was transiently increased at day 7, and then was significantly decreased at day 10 and 14 as compared with that in the control (Fig. 6C).

### Small animal PET imaging with [^18^F]FIMP

**Figure 7:**
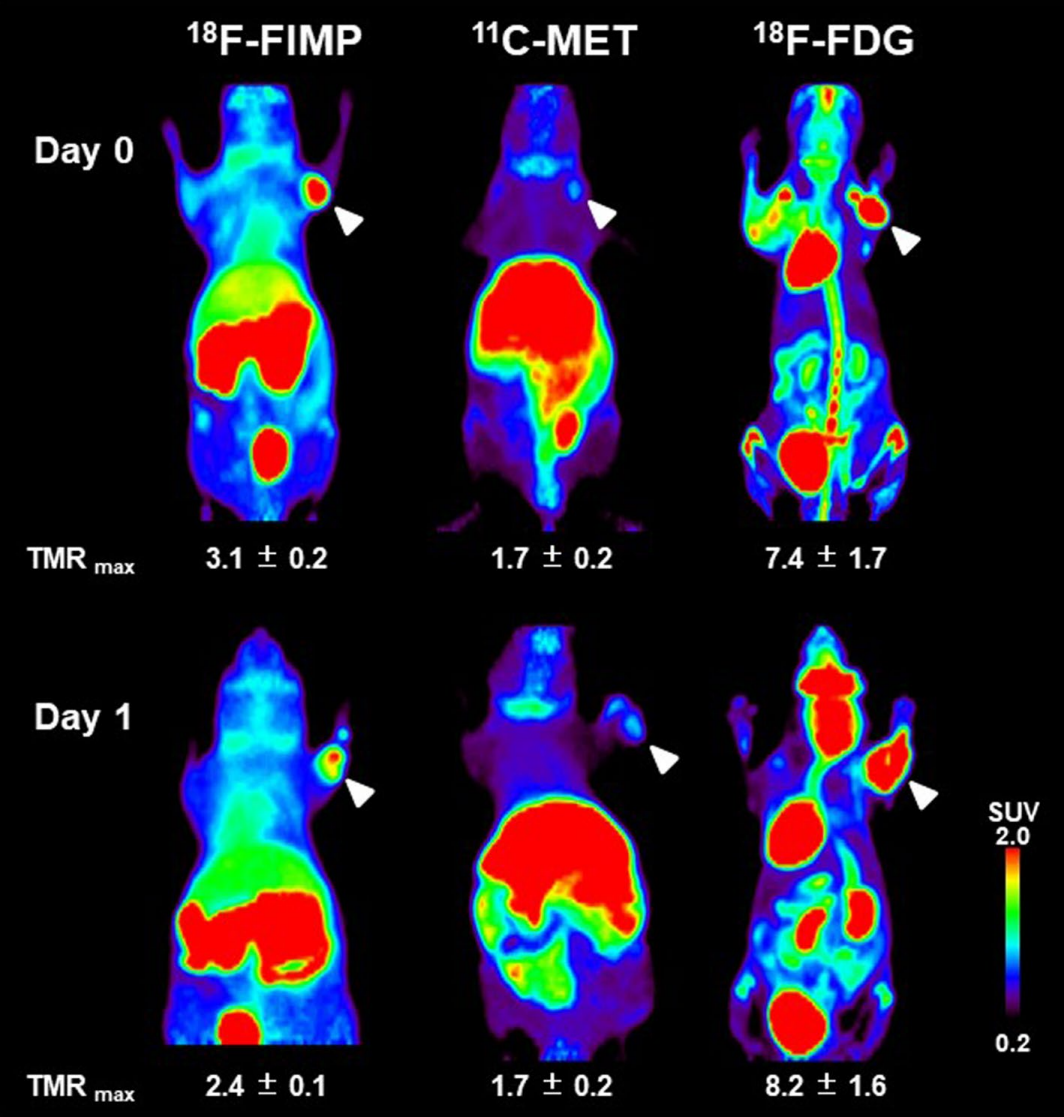
PET probe accumulation in LNZ308 tumors after irradiation. Images shown are the maximum intensity projection (MIP) image of [^18^F]FIMP, [^11^C]MET, and [^18^F]FDG-PET after irradiation on days 0 and 1. Tumors on right paws, as indicated by arrows, were irradiated on day 0 at the dose of 60 Gy. PET data were acquired 90 min after injection of [^18^F]FIMP and [^11^C]MET. [^18^F]FDG-PET data was acquired 55 to 100 min after injection. Data were reconstructed using OS-EM algorithm. Part of this figure was sourced from the following paper. Sci Rep. 2019 Oct 31;9(1):15718. 10.1038/s41598-019-52270-x.

Small animal PET imaging using the LNZ308 tumor-bearing mouse was performed on day 1 after irradiation (Fig. 7). Accumulation of [^18^F]FIMP was markedly decreased compared to that in the control (TMR: control, 3.1 ± 0.2; irradiation, 2.4 ± 0.1). However, accumulation of [^11^C]MET did not change compared to that in the control (TMR: 1.7 ± 0.2; 1.7 ± 0.2). Accumulation of [^18^F]FDG was slightly increased compared to that in the control (TMR: 7.4 ± 1.7; 8.2 ± 1.6). PET images of all observation points are shown in supplementary information (see Supplementary Figs. S1 –S3 online).

## Discussion

In this study, we revealed that [^18^F]FIMP could be more useful for early-phase evaluation of radiotherapy than the conventional probes [^11^C]MET and [^18^F]FDG.

We found that after irradiation, the tumor volume was significantly increased until day 11 and significantly decreased from day 18 compared to that before irradiation (day 0) (Fig. 1). These results suggest that anatomical changes such as decrease in tumor size due to irradiation is evident only from day 18 by anatomical imaging techniques such as CT or MRI, and before that, it is possible to detect biochemical changes only by using a functional imaging technologies such as PET. Based on this finding, our PET imaging studies were performed at days 1, 3, 7, 10, and 14 (arrow in Fig. 1).

Moreover, as a result of histological analysis of irradiated tissues, several characteristic changes in irradiation-induced cell death were observed in irradiated tumor tissue. Especially, it should be noted that morphologic and biochemical changes were already observed by H.E. staining (Fig. 2), Ki-67 (Figs. 3 and 4) and CD11b (Fig. 5) immunostaining just one day after irradiation. The decrease in Ki67 immunopositive cells reflects an increase in apoptotic cell death and is consequently associated with tumor shrinkage^24^. The frequency of CD11b-positive cells in tumors is highly correlated with COX-2 expression in tumors, reflecting the degree of radiation-induced inflammation^25^. That is, some biochemical changes may occur within the cells the next day after irradiation, suggesting that early-phase evaluation of radiotherapy is possible by using PET. As a confirmation of this hypothesis, the accumulations of [^11^C]MET and [^18^F]FDG did not change in the tumor tissues at the next day after irradiation, however, the accumulation of [^18^F]FIMP was significantly decreased at the next day after irradiation (Figs. 6, 7 and see Supplementary Fig. S1–S3 online). Our previous study identified that [^18^F]FIMP is a PET probe with sufficiently good affinity for LAT1 [28]. Therefore, LAT1 may be involved in this phenomenon. Previous studies have reported that Ki-67 and LAT1 expression in tumor tissues show a positive correlation^26–28^. Moreover, LAT1 is related to the regulation of cell proliferation by stimulating the mechanistic/mammalian target of rapamycin (mTOR) via the substrate L-leucine^29^. LAT1 expression after irradiation decreases in human brain tumor^30^. In this study, [^18^F]FIMP accumulation and Ki-67 expression in the tumor tissues after irradiation showed a similar tendency to decrease. Therefore, decrease in accumulation of [^18^F]FIMP after irradiation reflects a reduction of LAT1 expression, which may result in a decrease in cell proliferation.

The early-phase evaluation of tumor tissues after irradiation has already been studied with several PET probes such as [^18^F]FDG for glucose metabolism^31^, [^18^F]fluoromisonidazole (FMISO)^32^ and [^64^Cu]diacetyl-bis(*N*^4^-methylthiosemicarbazone) (ATSM)^33^ for hypoxia, [^11^C]MET^34^ for amino acid metabolism, [^18^F]fluorothymidine (FLT)^35^ for cell proliferation, and 2-(5-[^18^F]fluoro-pentyl)-2-methylmalonic acid (ML-10)^36^ for apoptosis. However, these probes are not sufficient to distinguish between tumor cells after irradiation and other changes such as inflammatory cells. In fact, immunoreactivity of CD11b, which is used as an inflammation marker, was appeared transiently increased at day 7 after irradiation (Fig. 5). Similarly, the accumulation of [^18^F]FDG was transiently increased at day 7 after irradiation (Fig. 6C).

In this study, only one dose of 60 Gy has been used as the radiation dose on the tumor tissue. However, in clinical radiation therapy, even lower dose or divided irradiation may be used to prevent the side effects of radiation. Therefore, in the next stage, we should test [^18^F]FIMP accumulation under these conditions.

Tumor uptake of [^18^F]FIMP but not of [^11^C]MET and [^18^F]FDG was effectively decreased 1 day after irradiation. Prognostic information after radiotherapy was obtained with [^18^F]FIMP at all time points during the first week after irradiation but only at limited time points with the 2 conventional probes [^11^C]MET and [^18^F]FDG. Based on these results, [^18^F]FIMP may be a PET probe involved in the early detection and prediction of radiotherapy efficacy, although further clarification is needed.

## Methods

### Probe synthesis

[^18^F]FIMP^22^ and [^11^C]MET^37^ were synthesized as described previously. All radiochemical purities of > 99.5% were determined by high performance liquid chromatography (HPLC). Human diagnostic grade [^18^F]FDG was provided by the Institute of Biomedical Research and Innovation (IBRI) hospital, Kobe, Japan.

### Animal model

All animal experimental protocols were approved by the RIKEN Animal Welfare Committee and were conducted in accordance with the National Institutes of Health Principles of Laboratory Animal Care. All applicable institutional and/or national guidelines for the care and use of animals were followed. All animal results are reported following ARRIVE guidelines.

The LNZ308 human glioblastoma cell line, which has been confirmed to express LAT1^22^, was a kind gift from Prof. Motoo Nagane of Department of Neurosurgery, Kyorin University, Japan. LNZ308 cells were cultured in Dulbecco's modified Eagle's medium (Nacalai Tesque, Inc., Kyoto, Japan) supplemented with 10% fetal bovine serum (Equitech-Bio, Inc., Kerrville, TX), 100 units/mL penicillin, and 100 μg/mL streptomycin (Nacalai Tesque, Inc.)^38^. LNZ308 cells were inoculated into the right forepaws of female BALB/cAJcl-nu/nu nude mice (CLEA Japan, Inc., Tokyo, Japan) via subcutaneous injection of 5 × 10^6^ cells in 100 μL Hank's balanced salt solution (Thermo Fisher Scientific, Waltham, MA)^38^. Tumor growth was monitored twice a week using a caliper.

### Radiation treatment

Irradiation was performed with an X-ray irradiation system (MX-160Labo, mediXtec Japan Corporation, Chiba, Japan). Mice whose tumor volume reached 100 mm^3^ were selected and irradiated with X-rays. The mice were restrained in polypropylene tubes under 1.5% isoflurane anesthesia. In order to prevent radiation damage to normal tissues, the tube was shielded with a barium sulfate containing sheet (thickness: 9.9 mm; GT—RS, Green Technologies, Tokyo, Japan) which was highly effective in preventing radiation and only the tumor tissue in the right forepaw was irradiated. The X-ray dose applied was 60 Gy at a tube voltage of 160 kV, a tube current of 2.95 mA, a dose rate of 1.2 Gy/min, and an irradiation time of 52 min.

### PET data acquisition and probe biodistribution

Mice were fasted for 14 h before [^18^F]FDG administration. [^18^F]FIMP and [^11^C]MET PET were administered to fed mice as a routine clinical [^11^C]MET PET examination. All mice were anesthetized with 1.5% isoflurane and placed on the bed of a microPET Focus 220 scanner (Siemens Medical Solutions USA, Inc., Knoxville, TN). Radiolabeled probes were dissolved in saline (0.1 mL) and administered via a cannula inserted into the tail vein. Emission data were acquired for 90 min after administration using a 3-dimensional (3D) list-mode method for [^18^F]FIMP and [^11^C]MET, and for 55–100 min after administration using a 3D list-mode method for [^18^F]FDG. Data were reconstructed using 2-dimensional filtered back projection (FBP) for quantification and a 2-dimensional ordered subset expectation maximization (OS-EM) algorithm for region of interest (ROI) definition. For ROI definition and further analysis, summed images (0–90 or 55–100 min post injection) were reconstructed. ROIs were drawn on several areas of tumor and muscle. Regional uptake of radioactivity in organs were decay-corrected based on injection times and expressed as the standardized uptake value (SUV), where SUV = tissue radioactivity concentration (MBq/cm^3^)/injected radioactivity (MBq) × body weight (g). Quantitative analysis of PET imaging data were also represented as tumor-to-muscle ratio (TMR)^22^. The Probe biodistribution study was performed using a different mouse than the PET study. After 90 min of probe administration, mice were sacrificed, and their organs quickly removed and washed with saline. Excised organs were weighed, and their radioactivity determined using a Wallac Wizard 1480 scintillation counter (PerkinElmer, Waltham, MA). Results were expressed as tumor-to-muscle ratio (TMR).

### Histological analysis

Tumor tissues were fixed in 4% paraformaldehyde for more than 24 h, embedded in paraffin, cut into 10-µm thick sections. And then sections were deparaffinized and rehydrated.

For immunostaining, the tissues were heated with 10 mM citric acid using a microwave for antigen retrieval, and then incubated in blocking buffer containing 10% goat serum (Vector Laboratories, Burlingame, CA) for 30 min, and further incubated at 4 °C overnight with rabbit polyclonal anti-Ki67 antibody (1/1000; Leica Biosystems, Nussloch, German) or rabbit monoclonal anti-CD11b antibody (1/1000; Abcam plc, Cambridge, UK) diluted with Tris Buffered Saline with Tween 20 (TBST). The sections were washed in TBST and then incubated for 30 min with horseradish peroxidase-labeled polymer conjugated anti-rabbit antibody (DAKO EnVision + , Agilent Technologies, Inc., Santa Clara, CA), followed by 3,3'-diaminobenzidine (DAB) (Nacalai Tesque) as substrate. Once the desired color developed, sections were lightly counterstained with Mayer’s hematoxylin (FUJIFILM Wako Pure Chemical Corporation, Tokyo, Japan).

For Hematoxylin–Eosin (H.E.) staining, the tissues were stained with Mayer’s hematoxylin and Eosin Y Solutions (FUJIFILM Wako Pure Chemical Corporation).

All slides were digitized and saved using SCN400 slide scanner (Leica Microsystems, Wetzlar, Germany).

For quantification of Ki-67 immuno-positive cells, the number of DAB- and hematoxylin-stained nuclei was counted using a public domain Java image processing program, ImageJ^39^. The DAB-stained nuclei were divided by the sum of DAB- and hematoxylin-stained nuclei and converted into a ratio of Ki-67 immuno-positive cells.

### Statistical analysis

Data are presented as mean ± standard deviation (SD). All statistical analyses were performed using Microsoft Excel 2010 version 14.0 (Microsoft, Redmond, WA) using Student’s t test. *P*-values less than 0.05 indicated significance.

### Ethical approval

All animal experimental protocols were approved by the Animal Care and Use Committee of RIKEN. The study is reported in this manuscript according to the ARRIVE guidelines.

## Supplementary Information

Supplementary Information.

## Data Availability

The datasets used and/or analysed during the current study available from the corresponding author on reasonable request.
