# Supplementary material for: Comparison of [18F]FIMP, [11C]MET, and [18F]FDG PET for early-phase assessment of radiotherapy response
Source: Sci Rep. 2023 Feb 3;13:1961. doi: 10.1038/s41598-023-29166-y (PMC9898523; doi:10.1038/s41598-023-29166-y)
Supplement: Supplementary file 1 — Supplementary Information. [file 41598_2023_29166_MOESM1_ESM.pptx]

## Slide 1
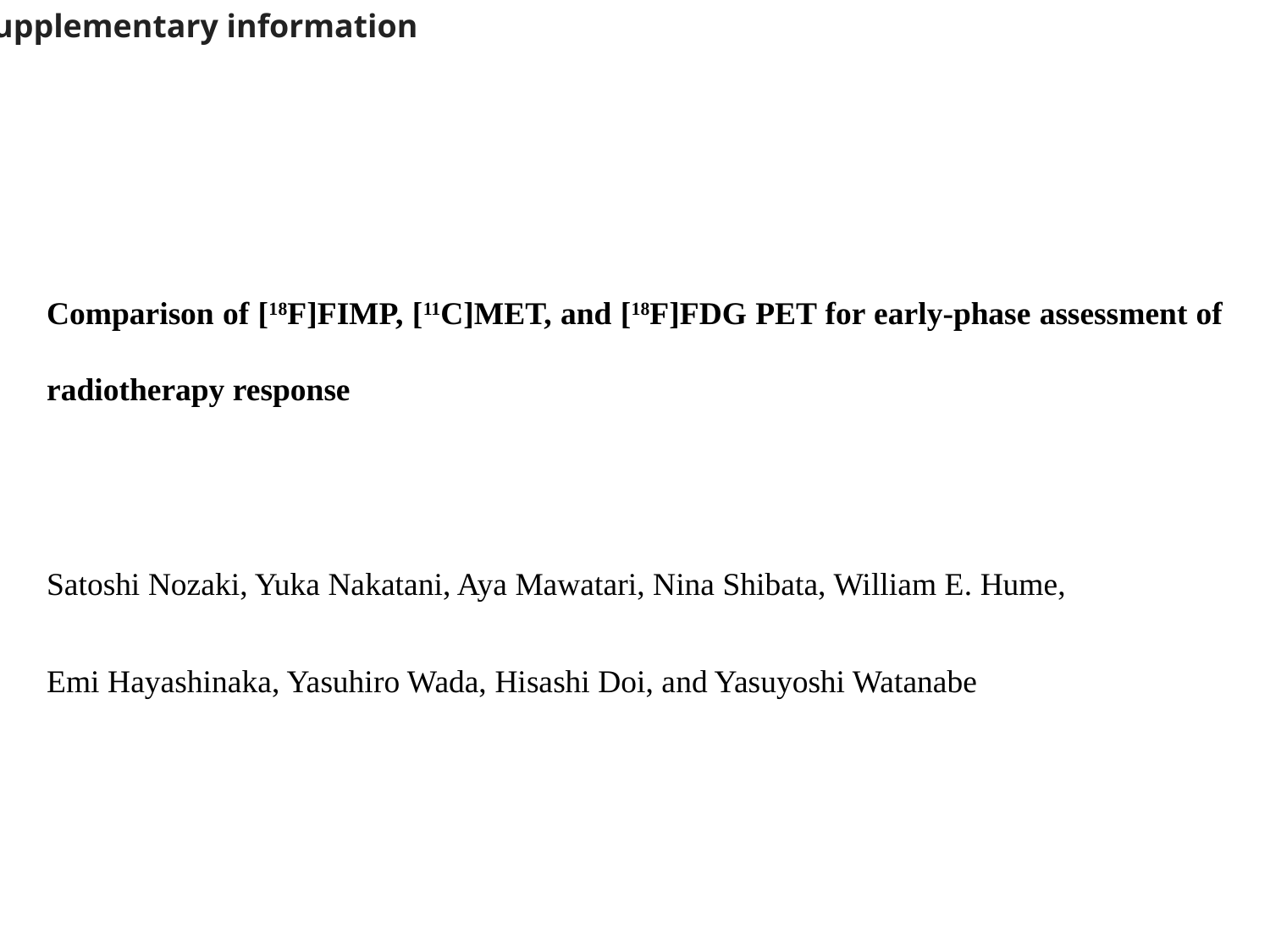

Supplementary information
Comparison of [18F]FIMP, [11C]MET, and [18F]FDG PET for early-phase assessment of radiotherapy response
Satoshi Nozaki, Yuka Nakatani, Aya Mawatari, Nina Shibata, William E. Hume,
Emi Hayashinaka, Yasuhiro Wada, Hisashi Doi, and Yasuyoshi Watanabe

## Slide 2
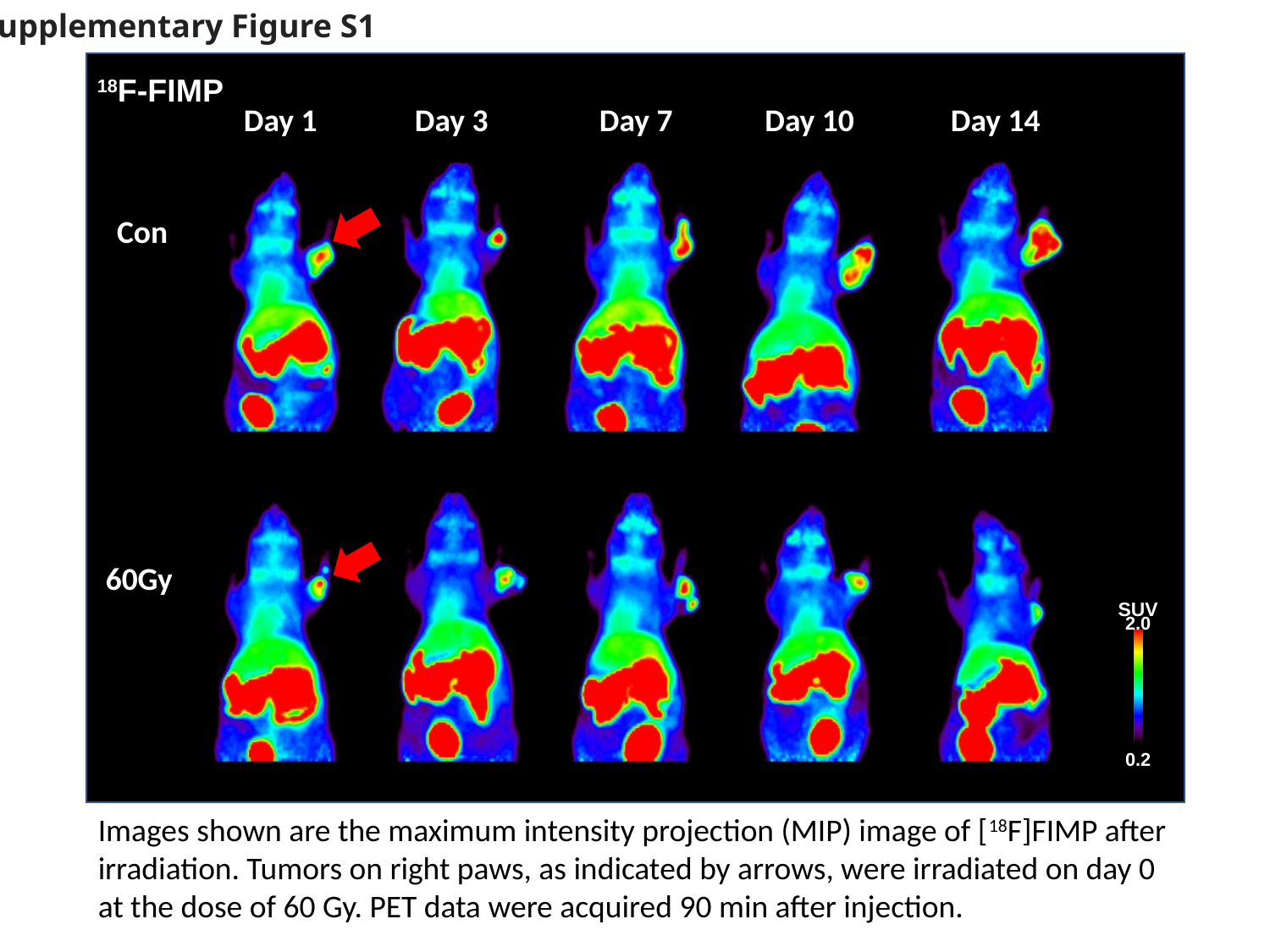

Supplementary Figure S1
18F-FIMP
Day 1
Day 3
Day 7
Day 10
Day 14
Con
60Gy
SUV
2.0
0.2
Images shown are the maximum intensity projection (MIP) image of [18F]FIMP after irradiation. Tumors on right paws, as indicated by arrows, were irradiated on day 0 at the dose of 60 Gy. PET data were acquired 90 min after injection.

## Slide 3
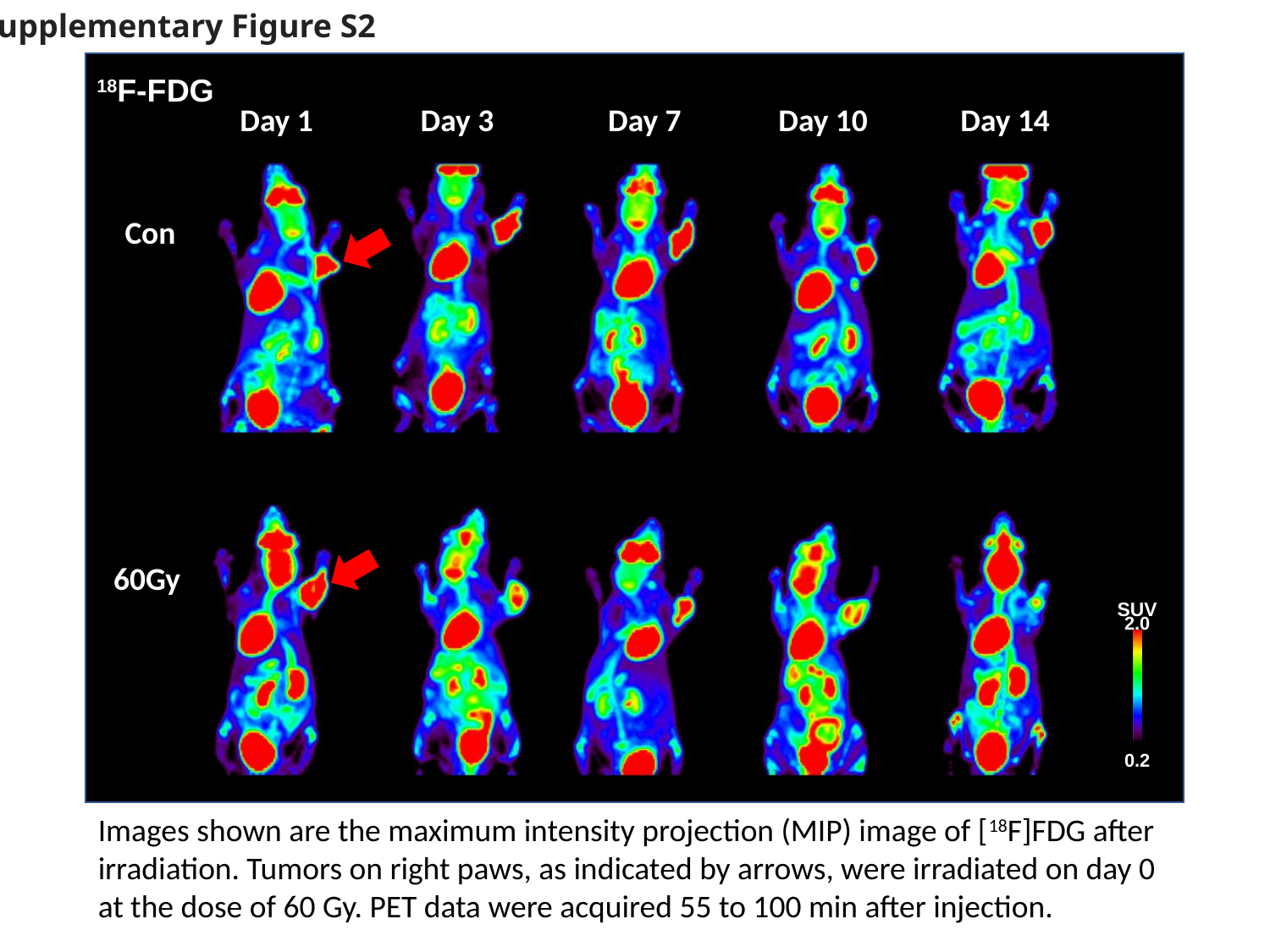

Supplementary Figure S2
18F-FDG
Day 1
Day 3
Day 7
Day 10
Day 14
Con
60Gy
SUV
2.0
0.2
Images shown are the maximum intensity projection (MIP) image of [18F]FDG after irradiation. Tumors on right paws, as indicated by arrows, were irradiated on day 0 at the dose of 60 Gy. PET data were acquired 55 to 100 min after injection.

## Slide 4
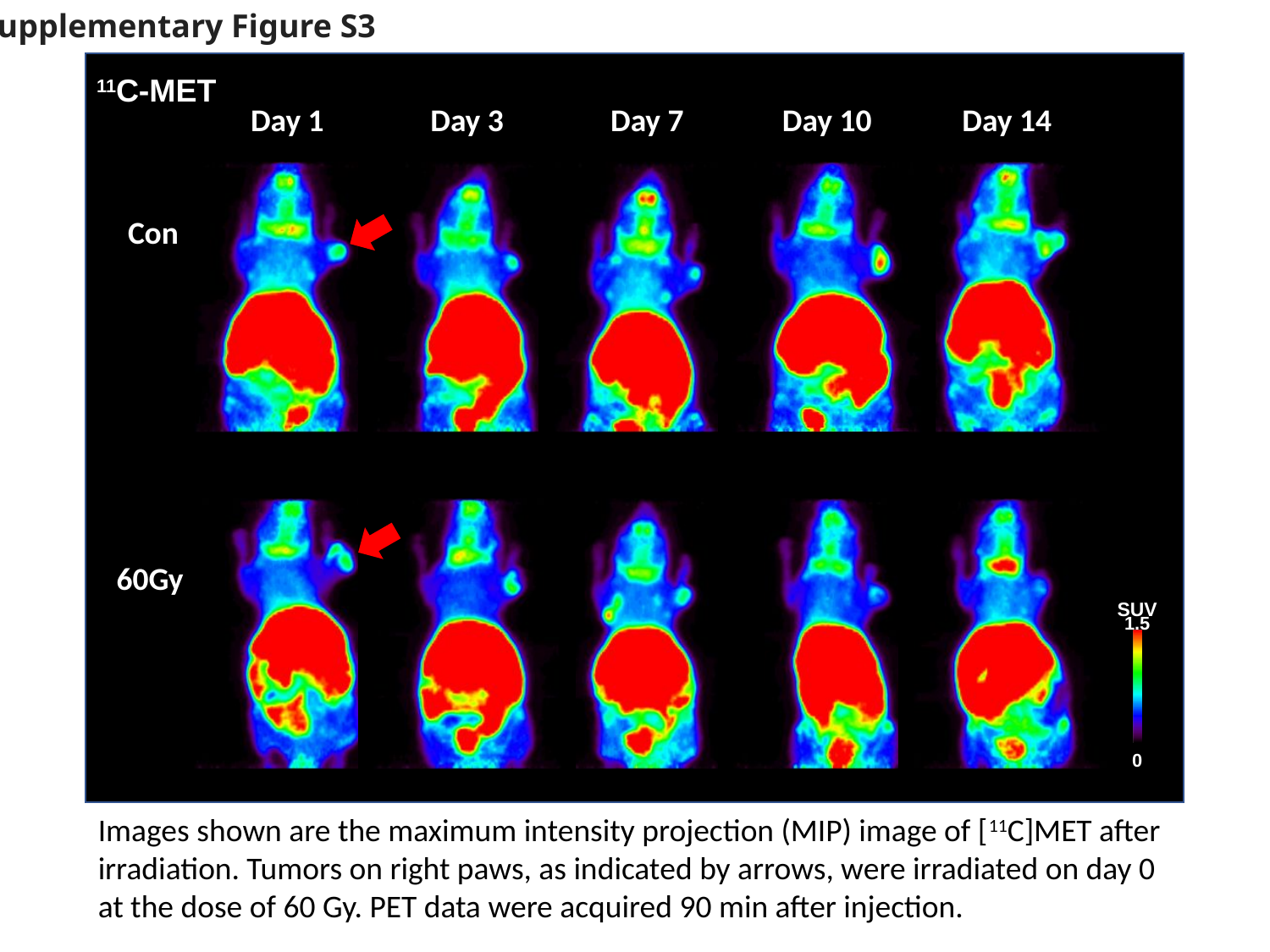

Supplementary Figure S3
11C-MET
Day 1
Day 3
Day 7
Day 10
Day 14
Con
60Gy
SUV
1.5
0
Images shown are the maximum intensity projection (MIP) image of [11C]MET after irradiation. Tumors on right paws, as indicated by arrows, were irradiated on day 0 at the dose of 60 Gy. PET data were acquired 90 min after injection.
